# Author Correction: Magnetically induced currents and aromaticity in ligand-stabilized Au and AuPt superatoms

**DOI:** 10.1038/s41467-022-28053-w

**Published:** 2022-01-20

**Authors:** Omar López-Estrada, Bernardo Zuniga-Gutierrez, Elli Selenius, Sami Malola, Hannu Häkkinen

**Affiliations:** 1 Department of Physics, Nanoscience Center, University of Jyväskylä, Jyväskylä, Finland; 2 Departamento de Química, Universidad de Guadalajara, CUCEI, Guadalajara, Jalisco Mexico; 3 Department of Chemistry, Nanoscience Center, University of Jyväskylä, Jyväskylä, Finland

**Keywords:** Computational chemistry, Method development, Nanoparticles

Correction to: *Nature Communications* 10.1038/s41467-021-22715-x, published online 30 April 2021.

The original version of this Article omitted to cite reference [23], which is relevant for a full understanding of the context of the previous work, in the final sentence of the first paragraph of the Introduction. The final sentence of the first paragraph of the introduction originally read “However, efficient self-consistent methods to calculate, analyse and visualize local MICs inside complex nanostructures have been lacking, preventing detailed analyses of...”. In the corrected version, the text: ‘after the pioneering work from Jusélius and colleagues [10]’, has been added to that sentence, and the word ‘preventing’ is substituted by ´limiting’. The correct version states: “However, after the pioneering work from Jusélius and colleagues [10], efficient self-consistent methods to calculate, analyse and visualize local MICs inside complex nanostructures have been lacking, limiting detailed analyses of…”. All the references following [10] have been renumbered incrementing by one.

The original version of this Article also contained an error in the first sentence of the Code Availability Statement, which originally read:

“Both DFT codes (GPAW and deMon2k) used in this work are open source and free to download for academic use at the respective sites”. In the corrected version, the text “open source and” has been removed. The correct version states: “Both DFT codes (GPAW and deMon2k) used in this work are free to download for academic use at the respective sites”.

This has been corrected in the PDF and HTML versions of the Article.

