# Supplementary material for: Magnetically induced currents and aromaticity in ligand-stabilized Au and AuPt superatoms
Source: Nat Commun. 2021 Apr 30;12:2477. doi: 10.1038/s41467-021-22715-x (PMC8087673; doi:10.1038/s41467-021-22715-x)
Supplement: Supplementary file 1 — Supplementary Information [file 41467_2021_22715_MOESM1_ESM.pdf]

Supplementary Information for: Magnetically induced currents and aromaticity in ligand-stabilized Au and AuPt superatoms

López-Estrada et al.

Supplementary Note 1

Electronic structure

To analyze the symmetries of the superatom orbitals, a Y_{lm} analysis[1] was performed for both **1** and **2** clusters (Figure 1). The three highest occupied orbitals of **1** and **2** show a P–type symmetry. In addition, both clusters have distinct HOMO-LUMO gaps, 1.88 eV (**1**) and 1.93 eV (**2**). Thus, the orbital analysis clearly indicates that the cluster cores $[HAu_9]^{2+}$ and $[HPtAu_8]^{+}$ have an eight-electron $1S^2 1P^6$ closed-shell configuration, c onsidering that each gold atom and the core-hydrogen contributes with one electron,[2] whereas the platinum and the phosphine ligands are neutral, is in good agreement with the electron counting rule.[1] While the HOMO-1 and HOMO-2 orbitals are degenerate in **1**, with HOMO 0.4 eV higher in energy, for cluster **2**, the energy differences between HOMO-2 and HOMO-1 and HOMO-1 and HOMO are 0.07 eV and 0.17 eV, respectively. The highest occupied orbitals in both clusters involve a contribution from the core-hydrogen. Similar P–type orbitals for **1** were obtained by previous DFT calculations[3] using PMe_3 as model ligands, however, the orbital involving the hydrogen contribution was HOMO-2, and the other two P–type orbitals were not degenerate. Likewise, P–type symmetry orbitals were observed in a $[HPdAu_8(PPh_3)_8]^{+}$ cluster with different energy order and $1S^2 1P^6$ superatomic closed-shell as **2**.[4]

For **1**, the LUMO is a superatomic orbital of D symmetry with most weight in the metal core, whereas for **2** the LUMO has more weight between the gold and phosphorus atoms, while still showing D symmetry in the PDOS analysis. The LUMOs do not show any contribution from the core-hydrogen.

Supplementary Figures

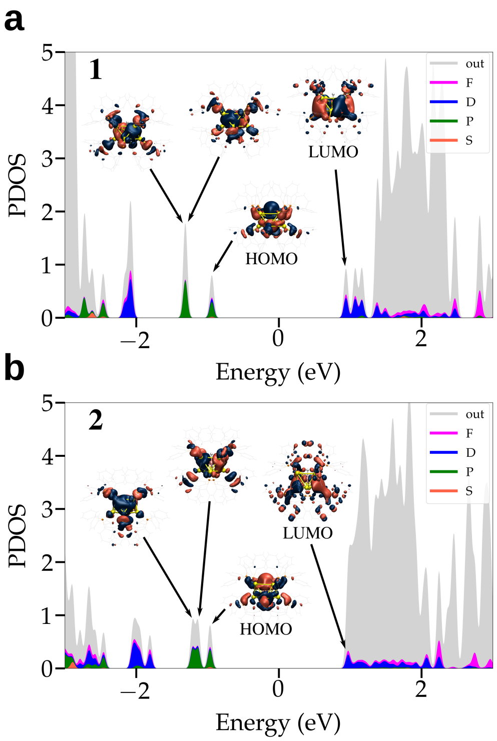

Supplementary Figure 1. Projected density of states. **a** $[HAu_9(PPh_3)_8]^{2+}$ and **b** $[HPtAu_8(PPh_3)_8]^{+}$. The frontier Kohn-Sham orbitals are shown at the isosurface value of 0.05 a.u.$^{-3/2}$

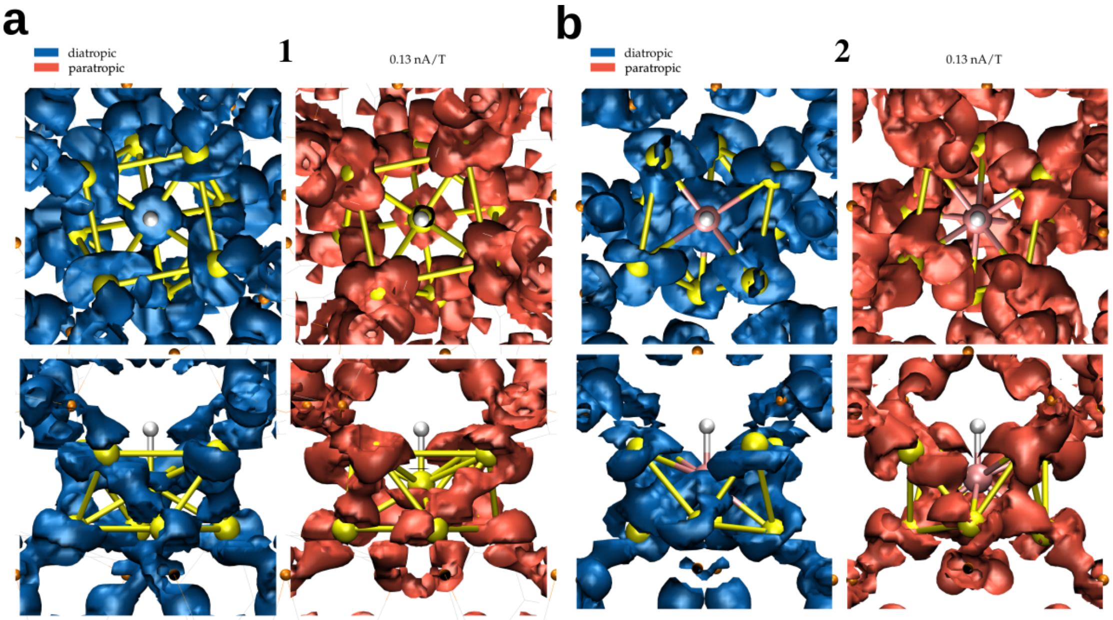

Supplementary Figure 2. 3D visualization of magnetically induced currents. 3D isovalue contours of the signed modulus of the magnetically induced current density at 0.13 nA/T for **a** $[HAu_9(PPh_3)_8]^{2+}$ (cluster **1**) and **b** $[HPtAu_8(PPh_3)_8]^{+}$ (cluster **2**). Diatropic (blue) and paratropic (red) contributions are indicated. Snapshots in z-direction (top row) and perpendicular to the x–y plane (bottom row).

Supplementary Tables

Supplementary Table 1. Experimental and DFT ^{1}H NMR chemical shift δ and standard deviation σ in ppm for clusters **1** and **2**.

		PBE/SDD		PBE/LANL2DZ		BP86/SDD		BP86/LANL2DZ	
**1**	Exp^a	δ	σ	δ	σ	δ	σ	δ	σ
o-proton	7.25	7.44	0.89	7.28	0.78	7.43	0.89	7.26	0.78
m-proton	6.62	7.27	0.39	7.21	0.38	7.26	0.39	7.16	0.38
p-proton	7.05	7.53	0.22	7.48	0.26	7.51	0.23	7.44	0.26
**2**	Exp^b	δ	σ	δ	σ	δ	σ	δ	σ
o-proton	7.29	7.68	1.07	7.56	1.02	7.68	1.07	7.54	1.02
m-proton	6.59	6.98	0.60	6.93	0.58	6.97	0.60	6.89	0.57
p-proton	7.02	7.24	0.22	7.19	0.23	7.23	0.22	7.14	0.23

^{a} Ref[3] was recorded in CD_2Cl_2 solution at 288 K; ^{b} Ref.[5] was recorded for a $[PtAu_8(PPh_3)_8]^{2+}$ cluster in CD_2Cl_2 solution at 298 K.

Supplementary Table 2. Experimental and DFT ^{1}H NMR chemical shift δ in ppm for the core-hydrogen in clusters **1** and **2**.

	Exp	PBE/SDD	PBE/LANL2DZ	BP86/SDD	BP86/LANL2DZ
**1**	15.1^a	3.88	2.98	3.96	2.99
**2**	5.4^b	-0.55	-1.36	-0.51	-1.38
Δ	9.7	4.43	4.34	4.47	4.37

^{a} Ref[3] ^{b} Ref[6,7]

Supplementary Methods

Gauge-Including Magnetically Induced Currents

In this section we formulate a mathematical expression for computing the electron current density within a molecule induced by an external magnetic field, $\mathbf{B}$. The derivation is based on the likeness of the **Biot-Savart law** with the ADFT-GIAO NMR shielding expressions,

$$\mathbf{B}^{ind} = -\frac{1}{c^2} \int \frac{\mathbf{r} - \mathbf{r}\,'}{|\mathbf{r} - \mathbf{r}\,'|^3} \times \mathbf{J}(\mathbf{r}\,')\, d\mathbf{r}\,' . \tag{1}$$

Atomic units are used throughout this text. The energy of a molecular system is altered due to the presence of the external magnetic field $\mathbf{B}$ and the field induced by the intrinsic magnetic moment of the nuclei $\boldsymbol{\mu}_C$. The energy change is so small that the molecular energy can be computed as a Taylor expansion,

$$E(\mathbf{B}, \{\mu_C\}) = E_0 + \mathbf{B} \cdot \frac{\partial E}{\partial \mathbf{B}} + \sum_{C=1}^{Nuclei} \boldsymbol{\mu}_C \cdot \frac{\partial E}{\partial \boldsymbol{\mu}_C} + \sum_{C=1}^{Nuclei} \mathbf{B} \cdot \boldsymbol{\sigma}_C \cdot \boldsymbol{\mu}_C + \ldots \tag{2}$$

The symbol $\boldsymbol{\sigma}_C$ can be interpreted as the magnetic shielding tensor at the position of the nucleus C. Thus, an element of this tensor is computed as a second order energy derivative in the limit $\mathbf{B}, \boldsymbol{\mu}_C \rightarrow 0$,

$$\sigma_{C,\lambda\eta} = \frac{\partial^2 E}{\partial B_\lambda \partial \mu_{C,\eta}} . \tag{3}$$

The same (second order) energy change can be expressed as a function of the electron current density, $\mathbf{J}(\mathbf{r}\,)$, and the magnetic vector potential associated to the magnetic moment of nucleus C, $\mathbf{A}_C(\mathbf{r}\,)$,

$$\Delta E^{(2)} = \sum_{C=1}^{Nuclei} \mathbf{B} \cdot \boldsymbol{\sigma}_C \cdot \boldsymbol{\mu}_C = -\sum_{C=1}^{Nuclei} \int \mathbf{A}_C(\mathbf{r}\,) \cdot \mathbf{J}(\mathbf{r}\,)\, d\mathbf{r} . \tag{4}$$

The shielding tensor can be also determined from the above definition by differentiating $\Delta E^{(2)}$ with respect to Cartesian components of the vectors $\boldsymbol{\mu}_C$ and $\mathbf{B}$. Firstly, for that purpose, the explicit form of the vector potential is introduced in the above definition,

$$\begin{aligned}
-\sum_{C=1}^{Nuclei} \int \mathbf{A}_C(\mathbf{r}) \cdot \mathbf{J}(\mathbf{r})\, d\mathbf{r} &= -\frac{1}{c^2} \sum_{C=1}^{Nuclei} \int \boldsymbol{\mu}_C \times \frac{\mathbf{r}-\mathbf{C}}{|\mathbf{r}-\mathbf{C}|^3} \cdot \mathbf{J}(\mathbf{r})\, d\mathbf{r} \\
&= -\frac{1}{c^2} \sum_{C=1}^{Nuclei} \int \boldsymbol{\mu}_C \cdot \frac{\mathbf{r}-\mathbf{C}}{|\mathbf{r}-\mathbf{C}|^3} \times \mathbf{J}(\mathbf{r})\, d\mathbf{r} \qquad (5) \\
&= -\frac{1}{c^2} \sum_{C=1}^{Nuclei} \sum_{\kappa,\alpha,\tau=x}^{z} \varepsilon_{\kappa\alpha\tau} \int \mu_{C,\kappa} \frac{\alpha - C_\alpha}{|\mathbf{r}-\mathbf{C}|^3} \mathcal{J}_\tau(\mathbf{r})\, d\mathbf{r}\,,
\end{aligned}$$

where $\mathbf{C}$ is the position vector for nucleus C and the Levi-Civita symbol is introduced. Differentation with respect to $\mu_{C,\eta}$ yields,

$$\frac{\partial \Delta E^{(2)}}{\partial \mu_{C,\eta}} = -\frac{1}{c^2} \sum_{\alpha,\tau=x}^{z} \varepsilon_{\eta\alpha\tau} \int \frac{\alpha - C_\alpha}{|\mathbf{r}-\mathbf{C}|^3} \mathcal{J}_\tau(\mathbf{r})\, d\mathbf{r}\,, \tag{6}$$

Now, the last expression is differentiating with respect to B_λ,

$$\sigma_{C,\lambda\eta} = \frac{\partial^2 \Delta E^{(2)}}{\partial B_\lambda \partial \mu_{C,\eta}} = -\frac{1}{c^2} \sum_{\alpha,\tau=x}^{z} \varepsilon_{\eta\alpha\tau} \int \frac{\alpha - C_\alpha}{|\mathbf{r}-\mathbf{C}|^3} \mathcal{J}_\tau^{(\lambda)}(\mathbf{r})\, d\mathbf{r}\,, \tag{7}$$

Here $\mathcal{J}_\tau^{(\lambda)}(\mathbf{r})$ is an element of the so-called **current density susceptibility tensor**:

$$\boldsymbol{\mathcal{J}}^{(\boldsymbol{\lambda})} = \begin{pmatrix} \mathcal{J}_x^{(x)} & \mathcal{J}_x^{(y)} & \mathcal{J}_x^{(z)} \\ \mathcal{J}_y^{(x)} & \mathcal{J}_y^{(y)} & \mathcal{J}_y^{(z)} \\ \mathcal{J}_z^{(x)} & \mathcal{J}_z^{(y)} & \mathcal{J}_z^{(z)} \end{pmatrix} \qquad \text{with} \qquad \mathcal{J}_\tau^{(\lambda)} \equiv \frac{\partial \mathcal{J}_\tau}{\partial B_\lambda}\,, \tag{8}$$

which is the first order correction to the total current density when it is expanded as a

Taylor series expansion,

$$\mathbf{J}(\mathbf{r}, \mathbf{B}^{ext}) \;=\; \mathbf{J}(\mathbf{r}, \mathbf{0}) \,+\, \boldsymbol{\mathcal{J}}^{(\boldsymbol{\lambda})}(\mathbf{r}, \mathbf{0}) \cdot \mathbf{B}^{ext} + ... \tag{9}$$

GIMIC within ADFT-GIAO framework

The working equations of the Auxiliary Density Functional Theory (ADFT) as implemented in the deMon2k software are now briefly presented. Within the ADFT framework the calculation of large systems is allowed (for a complete formulation of ADFT we refer the reader to[8]). The total ADFT energy[9] is given as

$$E^{ADFT} = \sum_{a,b} P_{ab} H_{ab} + \sum_{\bar{k}} \sum_{a,b} P_{ab} \left\langle \phi_a \phi_b || \bar{k} \right\rangle x_{\bar{k}} - \frac{1}{2} \sum_{\bar{k},\bar{l}} x_{\bar{k}} x_{\bar{l}} \left\langle \bar{k} || \bar{l} \right\rangle + E_{xc}[\tilde{\rho}]\,. \tag{10}$$

In this formula a and b denote basis functions, $\bar{k}$ and $\bar{l}$ auxiliary functions, $\boldsymbol{P}$ the density matrix, $\boldsymbol{H}$ the one-electron core matrix, $x_{\bar{l}}$ the fitting coefficients and $\tilde{\rho}$ the auxiliary density. The first term includes the kinetic, nuclear attraction and the magnetic interaction energy contributions; the second and third terms give account of the electron repulsion energy according to the variational fitting of the Coulomb potential approach (the symbol $||$ denotes the Coulomb operator $1/|\mathbf{r}_1 - \mathbf{r}_2\,|$), that introduces an approximated (auxiliary) density which is expressed as a linear expansion of auxiliary density (denoted by upper bar); finally, the last term is the exchange-correlation energy that is evaluated using the auxiliary density coming from the variational fitting of the Coulomb potential approach. The use of the auxiliary density introduces three center integrals instead of the four center ones in the repulsion energy contribution, that are the computational bottleneck in conventional DFT implementations, reducing in one order of magnitude the number of electron repulsion integrals to compute. The computational efficiency is also improved by the use of highly efficient recurrence relations.[10] Furthermore, the auxiliary density also reduces

in one order of magnitude the number of exchange correlation potential integrals that are evaluated by numerical methods. Both aspects confer a high computational efficiency to the ADFT approach.

The scheme known as the Gauge Including Atomic Orbitals (GIAO)[11] defined in the Coulomb gauge is used in combination with the ADFT framework with the aim of mitigating gauge origin problem (the value of the total energy and the magnetic property are not invariant to the election of the gauge origin, an unphysical behaviour). In the GIAO scheme, the basis functions are magnetic field (perturbation) dependent. They are built by attaching a London factor,[12] $\mathcal{L}_A$, to the normal basis function $a(\mathbf{r})$

$$\phi_a(\mathbf{r},\mathbf{B}) = \mathcal{L}_A\, a(\mathbf{r}) \qquad \text{with} \qquad \mathcal{L}_A = e^{-\frac{i}{2}\mathbf{B}\cdot(\mathbf{A}-\mathbf{G})\times\mathbf{r}}\,, \tag{11}$$

where $\mathbf{A}$ and $\mathbf{G}$ represent the position vectors of atom A, where $a(\mathbf{r})$ is centered, and of the gauge origin, respectively.

Performing the second order energy derivative in the ADFT-GIAO energy yields;

$$\sigma_{C,\lambda\eta} = \sum_{a,b} P_{ab}^{(\lambda)} H_{ab}^{(\eta)} + \sum_{a,b} P_{ab} H_{ab}^{(\lambda\eta)}\,. \tag{12}$$

The use of the greek letters λ and η as superscript indicates differentiation with respect to Cartesian components of the magnetic field and the nuclear magnetic moment, respectively. Here $\boldsymbol{P^{(\lambda)}}$ denotes the perturbed density matrix, while $H_{\mu\nu}^{(\eta)}$ and $H_{\mu\nu}^{(\lambda\eta)}$ represent first and second derivatives of an element of the core matrix, respectively. In the following, $\mu(\mathbf{r})$ and $\nu(\mathbf{r})$ refer to field independent atomic orbitals, then the first derivative of the core matrix

can be written as,

$$H^{(\eta)}_{\mu\nu} = -\frac{i}{c^2}\int \mu(\mathbf{r}\,)\left[\frac{\mathbf{r}-\mathbf{C}}{|\mathbf{r}-\mathbf{C}|^3}\times\nabla\right]_\eta \nu(\mathbf{r}\,)\,d\mathbf{r}\,; \tag{13}$$

and the second derivative as,

$$\begin{aligned} H^{(\lambda\eta)}_{\mu\nu} =& \frac{1}{2c^2}\int \mu(\mathbf{r}\,)\left[(\mathbf{A}-\mathbf{B})\times\mathbf{r}\,\right]_\lambda \left[\frac{\mathbf{r}-\mathbf{C}}{|\mathbf{r}-\mathbf{C}|^3}\times\nabla\right]_\eta \nu(\mathbf{r}\,)\,d\mathbf{r} - \\ & \frac{1}{2c^2}\int \mu(\mathbf{r}\,)\left[\frac{(\mathbf{r}-\mathbf{B})\cdot(\mathbf{r}-\mathbf{C})}{|\mathbf{r}-\mathbf{C}|^3}\delta_{\lambda\eta} - \frac{(\lambda-C_\lambda)(\eta-B_\eta)}{|\mathbf{r}-\mathbf{C}|^3}\right]\nu(\mathbf{r}\,)\,d\mathbf{r}\,. \end{aligned} \tag{14}$$

The calculation of $P^{(\lambda)}_{ab}$, the perturbed density matrix, is done via the McWeeny's equation[13]

$$P^{(\lambda)}_{ab} = 2\sum_o^{occ}\sum_u^{uno}\frac{\mathcal{K}^{(\lambda)}_{ou}-\mathcal{S}^{(\lambda)}_{ou}}{\varepsilon_o-\varepsilon_u}\left(c_{ao}c_{bu}-c_{au}c_{bo}\right) - \frac{1}{2}\sum_{d,e}P_{ad}S^{(\lambda)}_{de}P_{eb}\,, \tag{15}$$

where $\mathcal{K}^{(\lambda)}_{ou}$ and $\mathcal{S}^{(\lambda)}_{ou}$ are the perturbed Kohn-Sham and overlap matrices given in terms of molecular orbitals. The o denotes an occupied molecular orbital and u an unoccupied one, and the indices a, b, d, and e refer to atomic orbitals. The following expression permits to bring it in terms of atomic orbitals:

$$\mathcal{K}^{(\lambda)}_{ou} = \sum_{a,b} c_{ao}K^{(\lambda)}_{ab}c_{bu} \tag{16}$$

with $K^{(\lambda)}_{ab}$ defined as $K^{(\lambda)}_{ab} \equiv \lim_{\mathbf{B}\to 0}\frac{\partial K_{ab}}{\partial B_\lambda}$.
Within ADFT-GIAO, the expression for $K^{(\lambda)}_{ab}$ is

$$K^{(\lambda)}_{ab} = \sum_{a,b} H^{(\lambda)}_{ab} + \sum_{\bar{k}}\left\langle \phi^*_a\phi_b\,||\,\bar{k}\right\rangle^{(\lambda)}\left(x_{\bar{k}}+z_{\bar{k}}\right)\,. \tag{17}$$

The coefficients, $z_{\bar{k}}$ include the exchange correlation contributions and they are evaluated by means of numerical integration. These coefficients are computed during the SCF and stored in memory. As both the fitting and exchange correlation coefficients depend on the

auxiliary density that in turn is magnetic field (perturbation) independent, thus they are unaffected by the magnetic field differentiation. It means that the $z_{\bar{k}}$ are just read from memory and the ADFT-GIAO working equations for the magnetic shielding tensor are free of additional numerical integration improving the computational performance. In the conventional counterpart, the DFT-GIAO, the exchange correlation is evaluated using the so-called Kohn-Sham density, which is built with the GIAOs, introducing exchange correlation terms in $K_{ab}^{(\lambda)}$ that have not been previously calculated; thus, in the magnetic shielding working equations within the DFT-GIAO framework, additional numerical integration is needed. Working on the paramagnetic part of Eq. (12), we obtain

$$\begin{aligned}\sum_{\mu,\nu} P_{\mu\nu}^{(\lambda)} H_{\mu\nu}^{(\eta)} =& \sum_{\mu,\nu} P_{\mu\nu}^{(\lambda)} \int \mu(\mathbf{r}) \left[-\frac{i}{c^2} \frac{\mathbf{r}-\mathbf{C}}{|\mathbf{r}-\mathbf{C}|^3} \times \nabla \right]_\eta \nu(\mathbf{r})\, d\mathbf{r} \\ =& \sum_{\mu,\nu} P_{\mu\nu}^{(\lambda)} \int \mu(\mathbf{r}) \left(-\frac{i}{c^2} \right) \sum_{\alpha,\tau=x}^{z} \varepsilon_{\eta\alpha\tau} \frac{\alpha - C_\alpha}{|\mathbf{r}-\mathbf{C}|^3} \frac{\partial}{\partial\tau} \nu(\mathbf{r})\, d\mathbf{r} \\ =& -\frac{1}{c^2} \sum_{\alpha,\tau=x}^{z} \varepsilon_{\eta\alpha\tau} \int \frac{\alpha - C_\alpha}{|\mathbf{r}-\mathbf{C}|^3} \sum_{\mu,\nu} i\, P_{\mu\nu}^{(\lambda)}\, \mu(\mathbf{r}) \frac{\partial \nu(\mathbf{r})}{\partial\tau}\, d\mathbf{r}\,. \end{aligned} \tag{18}$$

Then from Eqs. (7) and (12) we can easily see, that the so-called paramagnetic contribution to Eq. (8) (labelled with superscript p) to the magnetic induced current is:

$$\mathcal{J}_\tau^{(\lambda),p} = i \sum_{\mu,\nu} P_{\mu\nu}^{(\lambda)}\, \mu(\mathbf{r}) \frac{\partial \nu(\mathbf{r})}{\partial\tau}\,. \tag{19}$$

We want to clarify that in the following equations $\mathbf{A}$ and $\mathbf{B}$ are the position vectors for atoms A and B from their respective GIAOs in Eq. (11) and $\mathbf{B}^{ext}$ refers to the external magnetic field. Working on the first diamagnetic part of Eq. (12),

$$
\begin{aligned}
\sum_{\mu,\nu} P_{\mu\nu} H_{\mu\nu}^{(\eta\lambda)} =& \sum_{\mu,\nu} P_{\mu\nu} \int \mu(\mathbf{r}\,) \frac{1}{2c^2} \left[(\mathbf{A}-\mathbf{B}) \times \mathbf{r}\,\right]_\lambda \left[\frac{\mathbf{r}-\mathbf{C}}{|\mathbf{r}-\mathbf{C}|^3} \times \nabla \right]_\eta \nu(\mathbf{r}\,)\, d\mathbf{r} \\
=& \sum_{\mu,\nu} P_{\mu\nu} \int \mu(\mathbf{r}\,) \left[(\mathbf{A}-\mathbf{B}) \times \mathbf{r}\,\right]_\lambda \left[\sum_{\alpha,\tau=x}^{z} \frac{\varepsilon_{\eta\alpha\tau}}{2c^2} \frac{\alpha - C_\alpha}{|\mathbf{r}-\mathbf{C}|^3} \frac{\partial}{\partial\tau} \right] \nu(\mathbf{r}\,)\, d\mathbf{r} \\
=& -\frac{1}{c^2} \sum_{\alpha,\tau=x}^{z} \varepsilon_{\eta\alpha\tau} \int \frac{\alpha - C_\alpha}{|\mathbf{r}-\mathbf{C}|^3} \sum_{\mu,\nu} \left(-\frac{1}{2} \right) P_{\mu\nu} \left[(\mathbf{A}-\mathbf{B}) \times \mathbf{r} \right]_\lambda \mu(\mathbf{r}\,) \frac{\partial \nu(\mathbf{r}\,)}{\partial\tau}\, d\mathbf{r}.
\end{aligned}
\tag{20}
$$

As previously analyzed for the paramagnetic part, we can find the tensor element for this first diamagnetic contribution to Eq. (8) (labelled with superscript $d1$):

$$
\mathcal{J}_\tau^{(\lambda),d1} = -\frac{1}{2} \sum_{\mu,\nu} P_{\mu\nu} \left[(\mathbf{A}-\mathbf{B}) \times \mathbf{r}\,\right]_\lambda \mu(\mathbf{r}\,) \frac{\partial \nu(\mathbf{r}\,)}{\partial\tau}. \tag{21}
$$

Working on the second part of the diamagnetic contribution, we get

$$
\begin{aligned}
\sum_{\mu,\nu} P_{\mu\nu} H_{\mu\nu}^{(\lambda\eta)} =& \sum_{\mu,\nu} P_{\mu\nu} \int \frac{1}{2c^2} \left[\frac{\mathbf{r}-\mathbf{C}}{|\mathbf{r}-\mathbf{C}|} \times \mathbf{B}^{ext} \times (\mathbf{r}-\mathbf{B}) \right]_\eta^{(\lambda)} \mu(\mathbf{r}\,)\nu(\mathbf{r}\,)\, d\mathbf{r} \\
=& \sum_{\mu,\nu} P_{\mu\nu} \int \sum_{\alpha,\tau=x}^{z} \frac{\varepsilon_{\eta\alpha\tau}}{2c^2} \frac{\alpha - C_\alpha}{|\mathbf{r}-\mathbf{C}|^3} \left[\mathbf{B}^{ext} \times (\mathbf{r}-\mathbf{B}\,) \right]_\tau^{(\lambda)} \mu(\mathbf{r}\,)\nu(\mathbf{r}\,)\, d\mathbf{r} \\
=& -\frac{1}{c^2} \sum_{\alpha,\tau=x}^{z} \varepsilon_{\eta\alpha\tau} \int \frac{\alpha - C_\alpha}{|\mathbf{r}-\mathbf{C}|^3} \sum_{\mu,\nu} \left(-\frac{1}{2} \right) P_{\mu\nu} [\mathbf{B}^{ext} \times (\mathbf{r}-\mathbf{B}\,)]_\tau^{(\lambda)} \mu(\mathbf{r}\,)\nu(\mathbf{r}\,)\, d\mathbf{r}.
\end{aligned}
\tag{22}
$$

Similarly as before, the contribution to the density current tensor due to the second diamagnetic contribution to Eq. (8) (labelled with superscript $d2$) is then expressed as

$$
\mathcal{J}_\tau^{(\lambda),d2} = -\frac{1}{2} \sum_{\mu,\nu} P_{\mu\nu} \left[\mathbf{B}^{ext} \times (\mathbf{r}-\mathbf{B}\,) \right]_\tau^{(\lambda)} \mu(\mathbf{r}\,)\nu(\mathbf{r}\,). \tag{23}
$$

For the sake of clarity, in the equation above the term in brackets is implicitly differentiated with respect to the magnetic field. After differentiation and applying the limit $\mathbf{B}^{ext} \to 0$, only the Cartesian component τ will remain from the cross product and the implementation is done as previously for the paramagnetic and first diamagnetic contribution.

Supplementary References

[1] Walter, M. *et al.* A unified view of ligand-protected gold clusters as superatom complexes. *Proc. Natl. Acad. Sci. U.S.A.* **105**, 9157–9162 (2008).

[2] Hu, G., Tang, Q., Lee, D., Wu, Z. & Jiang, D.-e. Metallic hydrogen in atomically precise gold nanoclusters. *Chem. Mater.* **29**, 4840–4847 (2017).

[3] Takano, S., Hirai, H., Muramatsu, S. & Tsukuda, T. Hydride-doped gold superatom $(Au_9H)^{2+}$: Synthesis, structure, and transformation. *J. Am. Chem. Soc* **140**, 8380–8383 (2018).

[4] Takano, S., Hirai, H., Muramatsu, S. & Tsukuda, T. Hydride-mediated controlled growth of a bimetallic $(Pd@Au_8)^{2+}$ superatom to a hydride-doped $(HPd@Au_{10})^{3+}$ superatom. *J. Am. Chem. Soc* **140**, 12314–12317 (2018).

[5] Takano, S., Ito, S. & Tsukuda, T. Efficient and Selective Conversion of Phosphine-Protected $(MAu_8)^{2+}$ (M = Pd, Pt) Superatoms to Thiolate-Protected $(MAu_{12})^{6+}$ or Alkynyl-Protected $(MAu_{12})^{4+}$ Superatoms via Hydride Doping. *J. Am. Chem. Soc* **141**, 15994–16002 (2019).

[6] Bour, J. *et al.* Hydride platinum gold clusters: synthesis and characterization. *Inorganica Chim. Acta* **181**, 195 – 200 (1991).

[7] Pignolet, L. H. *et al.* Phosphine-stabilized, platinum-gold and palladium-gold cluster compounds and applications in catalysis. *Coord. Chem. Rev.* **143**, 219 – 263 (1995).

[8] Calaminici, P. *et al.* *Auxiliary Density Functional Theory: From Molecules to Nanostructures*, 795–860 (Springer International Publishing, Cham, 2017).

[9] Köster, A. M., Reveles, J. U. & del Campo, J. M. Calculation of exchange-correlation potentials with auxiliary function densities. *J. Chem. Phys.* **121**, 3417–3424 (2004).

[10] Köster, A. M. Efficient recursive computation of molecular integrals for density functional methods. *J. Chem. Phys.* **104**, 4114–4124 (1996).

[11] Ditchfield, R. Molecular orbital theory of magnetic shielding and magnetic susceptibility. *J. Chem. Phys.* **56**, 5688–5691 (1972).

[12] London, F. Thèorie quantique des courants interatomiques dans les combinaisons aromatiques. *J. Phys. Radium* **8**, 397 (1937).

[13] McWeeny, R. *Methods of Molecular Quantum Mechanics* (Academic, London, 2001).
