# Supplementary material for: Magnetically induced currents and aromaticity in ligand-stabilized Au and AuPt superatoms
Source: Nat Commun. 2021 Apr 30;12:2477. doi: 10.1038/s41467-021-22715-x (PMC8087673; doi:10.1038/s41467-021-22715-x)
Supplement: Supplementary file 3 — Description of Additional Supplementary Files [file 41467_2021_22715_MOESM3_ESM.pdf]

Description of Additional Supplementary Files

File Name: Supplementary Data 1
Description: DFT-optimized coordinates of cluster 1

File Name: Supplementary Data 2
Description: DFT-optimized coordinates of cluster 2

File Name: Supplementary Movie 1
Description: 3D GIMIC animation of cluster 1

File Name: Supplementary Movie 2
Description: 3D GIMIC animation of cluster 2
